# Interaction between Vitamin D-Related Genetic Risk Score and Carbohydrate Intake on Body Fat Composition: A Study in Southeast Asian Minangkabau Women

**DOI:** 10.3390/nu13020326

**Published:** 2021-01-23

**Authors:** Buthaina E. Alathari, Arif Sabta Aji, Utami Ariyasra, Sri R. Sari, Nabila Tasrif, Finny F. Yani, Ikhwan R. Sudji, Julie A. Lovegrove, Nur I. Lipoeto, Karani S. Vimaleswaran

**Affiliations:** 1 Department of Food Science and Nutrition, Faculty of Health Sciences, The Public Authority for Applied Education and Training, Al Faiha 72853, Kuwait; B.E.A.A.Alathari@pgr.reading.ac.uk; 2 Hugh Sinclair Unit of Human Nutrition, Department of Food and Nutritional Sciences, University of Reading, Harry Nursten Building, Pepper Lane, Reading RG6 6DZ, UK; j.a.lovegrove@reading.ac.uk; 3 Department of Public Health, Alma Ata Graduate School of Public Health, University of Alma Ata, Yogyakarta 55183, Indonesia; sabtaaji@gmail.com; 4 Department of Nutrition, Faculty of Health Sciences, University of Alma Ata, Yogyakarta 55183, Indonesia; 5 Biomedical Science Department, Faculty of Medicine, Andalas University, West Sumatra 25172, Indonesia; tamiariyasra13@gmail.com (U.A.); srirahmasari03@gmail.com (S.R.S.); 6 Public Health Department, Faculty of Medicine, Andalas University, West Sumatra 25172, Indonesia; tasrif.nabila@gmail.com; 7 Department of Child Health, Faculty of Medicine, Andalas University, West Sumatra 25172, Indonesia; finny_fy@yahoo.com; 8 Department of Medical Laboratory Technology, Faculty of Health Science, University Perintis, Padang 25586, Indonesia; ir.sudji@gmail.com; 9 Department of Nutrition, Faculty of Medicine, Andalas University, West Sumatra 25172, Indonesia; indra.liputo@gmail.com

**Keywords:** genetic risk score, body fat percentage, Vitamin D, carbohydrate intake, Minangkabau women, Indonesia, metabolic disease

## Abstract

Metabolic diseases have been shown to be associated with low vitamin D status; however, the findings have been inconsistent. Hence, the objective of our study was to investigate the relationship between vitamin D status and metabolic disease-related traits in healthy Southeast Asian women and examine whether this relationship was modified by dietary factors using a nutrigenetic study. The study included 110 Minangkabau women (age: 25–60 years) from Padang, Indonesia. Genetic risk scores (GRS) were constructed based on five vitamin D-related single nucleotide polymorphisms (SNPs) (vitamin D-GRS) and ten metabolic disease-associated SNPs (metabolic-GRS). The metabolic-GRS was significantly associated with lower 25-hydroxyvitamin D (25(OH)D) concentrations (*p* = 0.009) and higher body mass index (BMI) (*p* = 0.016). Even though the vitamin D-GRS had no effect on metabolic traits (*p* > 0.12), an interaction was observed between the vitamin D-GRS and carbohydrate intake (g) on body fat percentage (BFP) (*p*_interaction_ = 0.049), where those individuals who consumed a high carbohydrate diet (mean ± SD: 319 g/d ± 46) and carried >2 vitamin D-lowering risk alleles had significantly higher BFP (*p* = 0.016). In summary, we have replicated the association of metabolic-GRS with higher BMI and lower 25(OH)D concentrations and identified a novel interaction between vitamin D-GRS and carbohydrate intake on body fat composition.

## 1. Introduction

Over a billion people in the world have vitamin D deficiency (VDD) and race and ethnicity have been shown to be strong predictors of vitamin D status, as measured by 25-hydroxyvitamin D (25(OH)D) concentrations. Low 25(OH)D concentrations have been shown to be associated with metabolic diseases, such as obesity and type 2 diabetes (T2D) [1]. Hereditary factors play a large role in VDD affecting up to 85% of serum concentrations of 25(OH)D [2,3] and the development of metabolic disorders [4,5,6,7,8,9,10,11,12]. However, we cannot overlook the effect of environment, lifestyle, nutrition, and dietary factors as major contributors to metabolic diseases [13,14]. Hence, it is of great importance to examine the lifestyle factors in distinct regions and varied environments in relation to their genetic susceptibility.

Indonesia is a country in Southeast Asia consisting of more than seventeen thousand islands that are split by the equator resulting in a tropical climate rich in sunlight with typically even temperatures year-round. Nevertheless, VDD rates are high in Indonesian women ranging between 60% to 95% [15]. Furthermore, incidence of obesity and T2D are high in Indonesia [16], with estimates of obesity and central obesity prevalence of 23.1% and 28%, respectively, based on data from Indonesia’s national health survey [17], and 10.3 million people are living with T2D, which constitutes as a major public health concern [18]. The prevalence of T2D has also been shown to be higher among Indonesian women compared to men (7.7 vs. 5.6%) [19,20].

Since only a few studies have examined the influence of single nucleotide polymorphisms (SNPs) on 25(OH)D levels in populations within Southeast Asia, our study focused on Minangkabau women from Padang, the capital of West Sumatra. The Minangkabau ethnic group is of particular interest given that the Minangkabau people have the largest matrilineal family structure in the world where the family line is inherited from the mother’s side and where women have higher status than men in the family and society [21,22]. We used a genetic study to investigate the association between vitamin D status and metabolic traits in a cohort of Minangkabau women from urban and rural areas of Padang and examined whether associations were modified by environmental and dietary factors using a nutrigenetic approach. The application of a genetic approach to establish the link between vitamin D status and metabolic diseases is favored over observational studies since genetic associations are less affected by confounding. Furthermore, we analyzed the combined effects of multiple genetic variants using two genetic risk scores (GRS) instead of the common single gene variant method in order to increase the statistical power to detect gene-diet interactions [23,24].

## 2. Methodology

### 2.1. Study Population

This study included 110 Indonesian women from the Minangkabau Indonesia Study on Nutrition and Genetics (MINANG), a cross-sectional study that was conducted in the city of Padang, Indonesia. The MINANG study is a part of the GeNuIne (Gene-Nutrient Interactions) Collaboration, the main aim of which is to investigate the impact of gene-nutrient interactions on cardiometabolic traits using population-based data from diverse ethnic groups [25,26]. The methodology has been published previously [21]. In brief, one hundred and thirty-three women were recruited from community health centers in two sub districts in Padang City: Padang Timur (urban area) and Kuranji (rural area). The inclusion criteria included: (i) healthy women, (ii) age between 25–60 years old, and (iii) Minangkabau ethnicity. After applying exclusion criteria (pregnancy or lactating, history of metabolic diseases or communicable disease, taking supplements, or being a relative to another participant), 117 women completed the MINANG study. For the present study, we excluded an additional seven participants, six had incomplete genetic data and one had incomplete dietary information.

The MINANG study was conducted in line with the principles of the Declaration of Helsinki and the study was approved by the Ethical Review Committee of the Medical Faculty, Andalas University (No.311/KEP/FK/2017). The study participants provided written informed consent prior to the start of the study.

### 2.2. Anthropometric Measures

Anthropometric measurements including height, weight, and waist circumference were obtained using standardized techniques. Body mass index (BMI) was calculated as weight (in kg) over height (in m^2^) and we used Asia-Pacific classification for defining obesity [27]. Body fat percentage (BFP) was assessed using Tanita MC780 (TANITA, Tokyo, Japan) multi-frequency segmental body composition analyzer.

### 2.3. Biochemical Measures

Fasting blood samples (5 mL) were taken, and serum was separated and stored at −20 °C until the assays were performed. Samples were analyzed for 25(OH)D, glucose, insulin, glycated hemoglobin A1c (HbA1c), total cholesterol, triglycerides, low density lipoprotein cholesterol (LDL-c), and high-density lipoprotein cholesterol (HDL-c). All biochemical assays were conducted using the Mark Microplate Spectrophotometer (Bio-Rad Laboratories Inc., Hercules, CA, USA). Serum 25(OH)D concentrations, and fasting glucose, insulin, and HbA1c were analyzed using enzyme-linked immunosorbent assay (ELISA) kits from Bioassay Technology Laboratory (Shanghai, China). The intra and inter-assay coefficients of variation for 25(OH)D were 5% and 8.1%, respectively. Fasting lipid levels were analyzed using enzymatic colorimetric procedures, namely glycerine phosphate oxidase peroxidase (GPO-PAP) for triglycerides, and cholesterol oxidase phenol 4-aminoantipyrine peroxidase (CHOD-PAP) for total cholesterol, and HDL-c. LDL-c was calculated using Friedewald’s formula.

### 2.4. Assessment of Dietary Intake

A proficient nutritionist collected dietary intake data from participants either at home or at an integrated health service post. For assessing dietary intake, a validated interviewer administered semi-quantitative food frequency questionnaire (SQ-FFQ) was used [28]. All data provided by participants were analyzed with Indonesian Food Database and Nutrisurvey (EBISpro, Willstätt, Germany) to estimate the total energy intake and macronutrient intakes.

### 2.5. SNP Selection and Genotyping

Five vitamin D-related SNPs were selected for investigation based on previously published studies that have shown association with 25(OH)D concentrations [29,30,31,32,33,34,35,36,37]: 7-dehydrocholesterol reductase (*DHCR7*) rs12785878 [30,35,36,37], 25-hydroxylase (CYP2R1) rs12794714 [29,37], 24-hydroxylase (*CYP24A1*) rs6013897 [33,35], vitamin D binding protein (DBP)/group-specific component (*GC*) rs2282679 [29,30], and calcium sensing receptor (CASR) rs1801725 [31,32,34]. Ten metabolic disease-related SNPs were selected based on previously published candidate gene studies and genome-wide association studies (GWAS) [4,5,6,11,38,39,40,41,42]: Fat mass and obesity-associated (*FTO*) SNPs rs8050136, rs9939609, and rs10163409 [5,40,41]; transcription factor 7-like 2 (*TCF7L2*) SNPs rs12255372 and rs7903146 [38]; melanocortin 4 receptor (*MC4R*) SNPs rs17782313 and rs2229616 [38,39]; potassium voltage-gated channel subfamily Q member 1 (*KCNQ1*) SNPs rs2237895 and rs2237892 [42]; cyclin dependent kinase inhibitor 2A/B (*CDKN2A*/*B*) SNP rs10811661 [11]. In this study, the minor alleles of the SNPs were in line with the minor alleles reported in the dbSNP for all the ethnic groups except for DHCR7 SNP rs12785878, where the minor allele is the “T” allele, which is in line with the South Asian and the East Asian populations as per the dbSNP, but, in the European population, the ‘G’ allele is the minor allele (Supplementary Table S1).

DNA was extracted from whole blood using the PureLink Genomic DNA Mini Kit (Invitrogen, Carlsbad, CA, USA). A NanoDrop spectrophotometer was used to determine DNA concentration. Genotyping was conducted using the competitive allele specific PCR-KASP^®^ assay at LGC Genomics, London, UK.

### 2.6. Statistical Analysis

**Figure 1:**
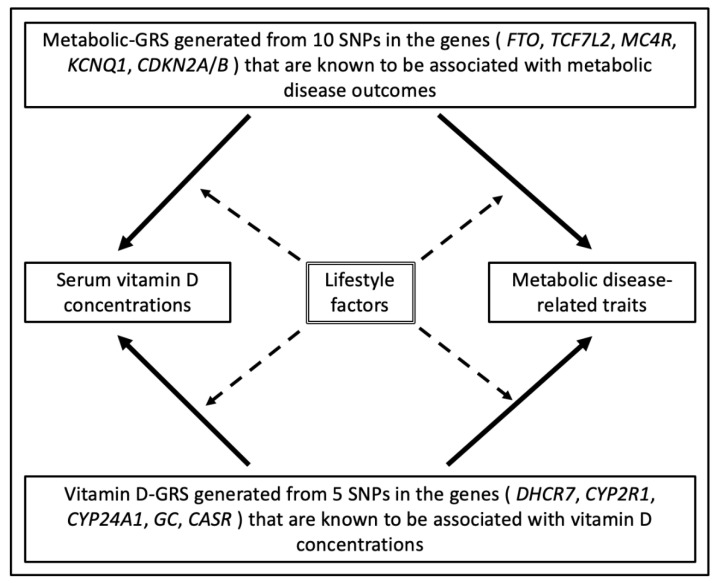
Study design. Genetic associations are represented by one-sided arrows with unbroken lines and interactions between GRS and diet on clinical and biochemical measurements are shown as one-sided arrows with broken lines. The association of the metabolic-GRS with 25(OH)D levels and metabolic traits and the association of vitamin D-GRS with 25(OH)D levels and metabolic traits were tested. In addition, the effect of dietary factors on these genetic associations was examined. Abbreviations: GRS: genetic risk score, SNP: single nucleotide polymorphism, *FTO*: fat mass and obesity-associated gene, *TCF7L2*: transcription factor 7-like 2 gene, *MC4R*: melanocortin 4 receptor gene, *KCNQ1*: potassium voltage-gated channel subfamily Q member 1, *CDKN2A*/*B*: cyclin dependent kinase inhibitor 2A/B, *DHCR7*: 7-dehydrocholesterol reductase, *CYP2R1*: 25-hydroxylase, *CYP24A1*: 24-hydroxylase, *GC*: group-specific component, *CASR*: calcium sensing receptor.

SPSS statistical software (v24; SPSS Inc., Chicago, IL, USA) was used to perform statistical analyses. All 15 gene variants were in the Hardy–Weinberg equilibrium (HWE) (*p* > 0.05), which was tested using a goodness-of-fit chi square test (Supplementary Table S1). Baseline characteristics (continuous variables) are presented as means ± standard deviations (SD) and comparisons between groups were tested using one-way analysis of variance (ANOVA). Test of normality was performed on all continuous variables and verified by the Shapiro–Wilk test. Log transformation was created for variables that were not normally distributed, which included age (years), waist circumference (WC) (cm), BFP (%), 25(OH)D level (ng/mL), glucose (mg/dL), HbA1c (ng/mL), insulin (nmlU/L), total cholesterol (mg/dL), HDL-c (mg/dL), LDL-c (mg/dL), triglycerides (mg/dL), total energy intake (Kcal), carbohydrate (g), protein (g), fat (g), fiber (g), saturated fatty acids (SFA) (g), monounsaturated fatty acids (MUFA) (g), and polyunsaturated fatty acids (PUFA) (g). Two separate Genetic risk scores (GRSs) were developed from the summation of the counts of the risk allele across each SNP. The vitamin D-related GRS was calculated from 5 SNPs and the metabolic disease-related GRS was calculated from 10 SNPs. A value of 0, 1, or 2 was assigned to each SNP, which denotes the number of risk alleles for each GRS. Then, these values were calculated by adding the number of risk alleles across each SNP. For each GRS, risk allele scores were then divided by the median and classified into a “low genetic risk group” and a “high genetic risk group.” Using the median of vitamin D- related GRS, low risk and high risk were classified as individuals carrying ≤2 (*n* = 67) and those carrying >2 (*n* = 43) risk alleles, respectively. For the metabolic disease-related GRS, low risk corresponds to individuals carrying <4 (*n* = 54) and high risk corresponds to those carrying ≥4 (*n* = 56). Figure 1 represents the study design of the analyses performed.

The effect of both the vitamin D-related GRS and the metabolic disease-related GRS on anthropometric and biochemical outcomes (BMI, WC, BFP, 25(OH)D, glucose, HbA1c, fasting insulin, total cholesterol, HDL-c, LDL-c, and triglycerides) were analyzed using linear regression models. Furthermore, the interaction between GRSs and dietary factors on clinical and biochemical variables were tested using linear regression models by including the interaction term (GRS*dietary factor) in these models. Models were adjusted for age, BMI, location (rural or urban), and total energy intake, wherever appropriate. Dietary factors included carbohydrate, protein, fat, and fiber intake in grams. Statistically significant interactions were examined further where study participants were stratified by the tertiles of dietary consumption. Power calculations were not performed as there are no studies in relation to GRS and vitamin D status and no previously reported effect sizes are available for the Southeast Asian populations.

## 3. Results

### 3.1. Characteristics of Participants

A total of 110 women (mean age 40.46 ± 9.38 years) were included, where 51% were from an urban area (Padang Timur) and 49% were from a rural area (Kuranji) in Padang, Indonesia. In this study, 20% of the women were vitamin D deficient and 40% were vitamin D insufficient (Table 1). There was a significant difference in the mean age across the three vitamin D groups (*p* = 0.001) where women with mean age 34.3 ± 10.3 years were vitamin D deficient and women with mean age 43.8 ± 7.8 years were vitamin D sufficient. There were no significant differences in the clinical and biochemical parameters across the three groups classified based on vitamin D status (Table 1).

### 3.2. Association between Metabolic-GRS and Anthropometric and Biochemical Measurements

**Figure 2:**
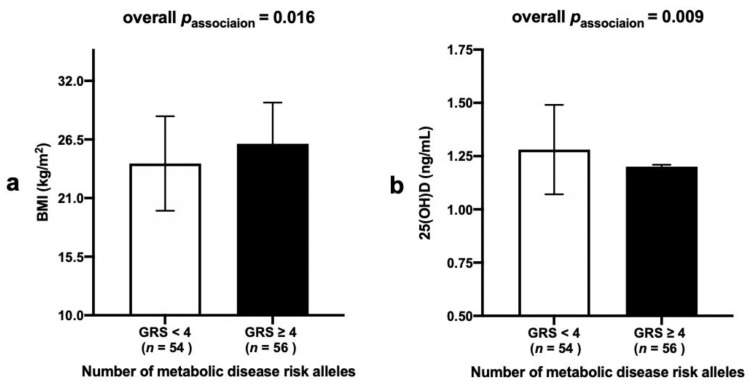
(**a**) Association between metabolic-GRS and BMI in Indonesian women. Those who carried ≥4 metabolic risk alleles had higher BMI compared to individuals with <4 risk alleles (*p* = 0.016). (**b**) Association between metabolic-GRS and 25(OH)D concentrations in Indonesian women. Those who carried ≥4 metabolic risk alleles had lower 25(OH)D concentrations compared to individuals with <4 risk alleles (*p* = 0.009). Abbreviations: GRS: genetic risk score, BMI: body mass index, 25(OH)D: 25-hydroxyvitamin D.

A significant association was found between the metabolic-GRS and BMI (*p* = 0.016), where participants who carried ≥4 risk alleles had higher BMI levels (mean ± SD: 26.10 ± 3.87) compared to those with <4 risk alleles (mean ± SD: 24.25 ± 4.43), Figure 2a. Moreover, a significant association between the metabolic-GRS and serum 25(OH)D concentrations (*p* = 0.009) was observed where individuals who carried ≥4 risk alleles had lower 25(OH)D levels (mean ± SD: 1.20 ± 0.19) compared to those with <4 risk alleles (mean ± SD: 1.28 ± 0.21), Figure 2b.

### 3.3. Association between Vitamin D-GRS and Anthropometric and Biochemical Measurements

There was no statistically significant association between the vitamin D-GRS and 25(OH)D levels (*p* = 0.93). Furthermore, there was no significant association of the clinical and biochemical characteristics such as BMI, WC, BFP, glucose, HbA1c, insulin, total cholesterol, HDL-c, LDL-c, and triglycerides with vitamin D-GRS (*p* > 0.12 for all comparisons) (Supplementary Table S2).

### 3.4. Interaction between the Vitamin D-GRS and Dietary Factors on Biochemical and Anthropometric Parameters

**Figure 3:**
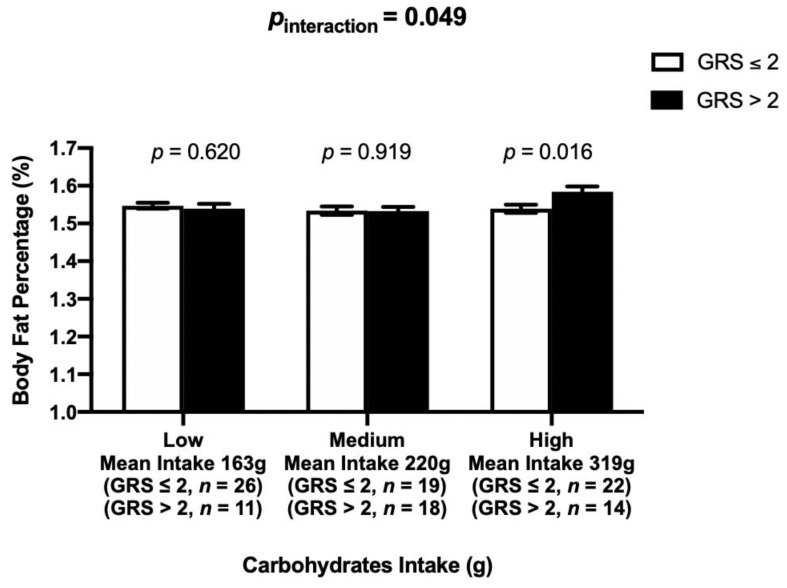
Interaction between the vitamin D-GRS and dietary carbohydrate intake (g) on BFP (%) (*p*_interaction_ = 0.049). Those who were on the highest tertile of carbohydrate intake and carried >2 risk alleles had significantly higher BFP compared to individuals carrying ≤2 risk alleles (*p* = 0.016). Abbreviations: GRS: genetic risk score, BFP: body fat percentage.

There was a statistically significant interaction between the vitamin D-GRS and carbohydrate intake on log BFP (*p*_interaction_ = 0.049), where participants who consumed high amounts of carbohydrates (mean ± SD: 319 g/d ± 46) and carried >2 risk alleles (mean ± SD: 1.60 ± 0.04, *p* = 0.016) had significantly higher log BFP than those with ≤2 risk alleles (mean ± SD: 1.53 ± 0.11, *p* = 0.016), Figure 3.

### 3.5. Interaction between the Metabolic-GRS and Dietary Factors on Clinical and Biochemical Characteristics

No statistically significant interactions were found between metabolic-GRS and dietary intake on serum 25(OH)D concentrations and clinical and biochemical parameters (*p* > 0.997 for all comparisons), (Supplementary Table S3).

## 4. Discussion

To date, this is the first study to utilize a nutrigenetic approach to examine the relationship between vitamin D status and metabolic traits in Southeast Asians. Our study has confirmed the association of metabolic-GRS with higher BMI and lower 25(OH)D concentrations. Additionally, the study has shown an impact of genetically instrumented vitamin D status on BFP, a marker of body fat composition, through the influence of dietary carbohydrate intake, where women consuming high amounts of carbohydrates (mean ± SD: 319 g/d ± 46) and those with higher genetic risk of VDD had higher BFP. Given that percent body fat is a better predictor of cardiovascular risk factors than BMI, if the results are replicated in future studies, our findings may have a significant public health implication in preventing cardiovascular diseases in Minangkabau women by developing dietary intervention strategies to reduce the intake of carbohydrates.

In the present study, a metabolic-GRS consisting of 10 SNPs known to be associated with obesity and type 2 diabetes [4,5,6,11,38,39,40,41,42] was created; this GRS was used as a genetic instrument to explore the link between metabolic disease-related traits and vitamin D status. The study showed that participants carrying ≥4 metabolic risk alleles had higher BMI and lower 25(OH)D concentrations compared to those carrying <4 risk alleles. There are several mechanisms that have been proposed for the effect of obesity on vitamin D status, such as, vitamin D dilution due to increased fat stores, having more vitamin D within adipose tissue, and lifestyle variations between obese and lean individuals, as well as differences in the activity of the vitamin D activating enzymes between obese and lean individuals [44]. Furthermore, a Mendelian Randomization (MR) study in ~42,024 Caucasians had demonstrated a causal relationship between obesity and vitamin D status, where a 10% higher genetically instrumented BMI was associated with 4.2% lower 25(OH)D concentrations [45]. These findings suggest that increased obesity can lead to lower vitamin D status, which was also confirmed in the present study where a significant association between metabolic-GRS and lower 25(OH)D concentrations was observed.

Vitamin D-GRS was constructed as a genetic instrument for vitamin D status using five common vitamin D pathway-related SNPs that have been identified by the GWA scans [35,46,47,48,49]. However, our study did not find any significant association of vitamin D-GRS with 25OHD concentrations and other clinical and biochemical parameters. The lack of association implies that linear decreases in vitamin D may not have an impact on the metabolic disease-related outcomes, which is in line with the findings from the MR study [45]. Furthermore, a genetic association study in two large European cohorts (*n* = 5224, 123,865; respectively) failed to demonstrate a significant impact of vitamin D-related gene variants on obesity traits, suggesting that vitamin D pathway genes are unlikely to have an important impact on the obesity-related outcomes [50]. Animal studies have convincingly illustrated the role of the vitamin D genes in contributing to adiposity phenotypes [51,52]; however, it is possible that there is no direct effect of 25(OH)D concentrations on adiposity in humans.

Even though there was no association of vitamin D-GRS with metabolic disease risk, there was an interaction of vitamin D-GRS with dietary carbohydrate intake on BFP, where those who consumed a high carbohydrate diet and had high genetic risk of VDD had significantly higher BFP than those with low genetic risk. The total carbohydrate intake of our study participants ranged from 121–436 g/day (mean ± SD: 233.08 ± 71.34) and there was no significant difference in carbohydrate intake between those living in urban (mean ± SD: 238.74 ± 77.35) and rural (mean ± SD: 227.21 ± 64.73) areas (*p* = 0.40). The intake of carbohydrates in the highest tertile (mean ± SD: 319 ± 46 g/d) is above the Indonesian dietary guidelines [53,54], which recommends consuming 50% of total energy from carbohydrates. In line with these findings, a recent study [38] from our team in 545 Asian Indians had also demonstrated an interaction of metabolic-GRS with dietary carbohydrate intake on 25(OH)D concentrations, where individuals consuming a low carbohydrate diet (≤62%, equivalent to ~346 g/d) and having lower metabolic genetic risk had significantly higher concentrations of 25(OH)D. Increased carbohydrate intake, in particular refined carbohydrates and sugars, have been linked to obesity and T2D [55], and the use of restricted carbohydrate diets have shown to be effective in reducing body weight and WC, as well as, fasting glucose, HbA1c, and plasma insulin levels [56,57]. The interaction of vitamin D-GRS with dietary carbohydrate intake on BFP that was observed in our study is biologically possible, given that vitamin D has been shown to mediate the impact of reduced consumption of carbohydrate through its direct action on pancreatic beta-cell function [58,59]. While studies have shown that there might be a failure to expand beta-cell mass in response to obesity [60], it is possible that lower levels of vitamin D due to genetic susceptibility can lead to obesity in the presence of high carbohydrate diet. However, the mechanism by which the carbohydrates interact with vitamin D- related genetic variants and affect body composition is unclear and requires further exploration. Given that the main source of energy for the Minangkabau is carbohydrates, where rice, banana, cassava, corn, sweet potato, sago, noodles, glutinous rice, and mung bean are part of their daily meals [61], our findings will have public health relevance if replicated in larger cohorts.

BMI is the most common indicator of obesity; however, BMI does not distinguish between lean mass and adiposity. Given that BFP can distinguish between fat and lean body mass [62] and increased BFP has been shown to be associated with cardiometabolic diseases and mortality [63], BFP is considered as a better indicator of obesity. Thus, detecting excess adiposity and body fat composition has become particularly important to make accurate conclusions with regards to establishing causal relationships and identifying risk to prevent the onset of metabolic and cardiovascular diseases [62,63]. There are ethnic differences in body fat composition as we explore the complex interaction between the genes, lifestyle, and culture [21]. Understanding of ethnic differences may lead to the implementation of effective approaches to recognize and prevent metabolic diseases across different ethnic groups [64]. It is important that the findings from this study are replicated before consideration is given to personalized dietary advice for Indonesian women carrying a higher genetic risk of VDD.

The current study has strengths including being the first nutrigenetic study to evaluate the relationship of vitamin D status with metabolic disease risk in Southeast Asians. The construction of the GRSs instead of a single SNP approach increases the statistical power to identify gene-diet interactions [23,24]. Additionally, the use of a comprehensive, validated SQ-FFQ [28] collected by a trained nutritionist increases the accuracy of dietary data collection. The study does have several limitations that should be acknowledged. The sample size of the study is small; however, the study was sufficiently powered to identify significant associations and gene-diet interactions. Even though we used a validated SQ-FFQ, bias due to self-reported dietary intake information cannot be excluded. The study did not include data on specific categories of foods that were included in the total carbohydrate count and it did not quantify different classes of carbohydrates, such as complex carbohydrates and simple disaccharides and monosaccharides. Finally, the study was limited to Minangkabau women, and hence, our findings cannot be generalized to the Indonesian population.

## 5. Conclusions

In summary, our study has replicated the association of metabolic-GRS with higher BMI and lower 25(OH)D concentrations and identified a novel interaction of vitamin D-GRS with carbohydrate intake on BFP, where women with increased genetic risk of vitamin D deficiency and who consume higher amounts of carbohydrates have increased body fat composition. This finding is potentially valuable in introducing nutritional recommendations to reduce carbohydrate intake for Indonesian women belonging to the Minangkabau community, as a third of our study participants had a mean intake of 319 g/d. This is equivalent to 71.8% dietary intake of carbohydrates, based on the average intake of our participants of 1776 kcal/day, which is very high compared to the Indonesian dietary guidelines that recommends obtaining 50% of total energy from carbohydrates [53,54]. However, before dietary interventions can be developed and recommended to improve vitamin D status and reduce carbohydrate intake in Indonesian women, replication of our findings in a larger cohort is required.

## Figures and Tables

**Table 1:** Anthropometric and biochemical characteristics of the study participants stratified based on vitamin D status.

Characteristics of Study Participants	Vitamin D Sufficiency (≥20 ng/mL) (*n* = 44)	Vitamin D Insufficiency (≥12 ≤ 19 ng/mL) (*n* = 44)	Vitamin D Deficiency <12 ng/mL (*n* = 22)	*p* Value
Age (years)	43.8 ± 7.8	40.0 ± 10.9	34.3 ± 10.3	0.001
BMI (kg/m^2^)	25.7 ± 4.4	25.3 ± 4.0	24.1 ± 4.3	0.358
WC (cm)	86.5 ± 9.9	82.6 ± 11.3	81.0 ± 8.6	0.070
BFP (%)	36.2 ± 7.8	36.4 ± 6.5	34.0 ± 6.3	0.392
Glucose (mg/dL)	96.1 ± 30.4	92.1 ± 9.0	86.4 ± 10.6	0.198
HbA1c (ng/mL)	710 ± 703	670 ± 573	440 ± 209	0.178
Fasting Insulin (nmlU/L)	36,020 ± 29,916	32,915 ± 24,107	22,261 ± 8092	0.101
Total Cholesterol (mg/dL)	209 ± 46	216 ± 44	194 ± 37	0.142
HDL Cholesterol (mg/dL)	57.4 ± 8.4	59.9 ± 11.4	61.2 ± 11.4	0.295
LDL Cholesterol (mg/dL)	127.3 ± 40.3	135.4 ± 41.9	113.8 ± 31.8	0.115
Serum Triglycerides (mg/dL)	103 ± 44.9	93.8 ± 41.7	96.7 ± 44.0	0.601
Total Energy Intake (kcal)	1797.0 ± 645.0	1695.0 ± 545.0	1894.3 ± 675.4	0.442
Carbohydrate (g)	233.7 ± 75.1	225.9 ± 58.9	246.0 ± 86.5	0.563
Protein (g)	77.2 ± 41.7	74.2 ± 30.4	82.2 ± 39.2	0.707
Fat (g)	61.2 ± 36.1	54.7 ± 31.6	64.1 ± 32.1	0.498
Dietary Fibre (g)	8.6 ± 4.3	8.3 ± 3.5	10.2 ± 5.5	0.218

Data are presented as means ± SD. *P* values were calculated by using one-way analysis of variance (ANOVA). Vitamin D cut-off points were based on the suggestions of the Institute of Medicine for vitamin D levels [43]. Abbreviations: BMI, body mass index; WC, waist circumference; BFP, body fat percentage; 25(OH)D, 25-hydroxyvitamin D; HbA1C, glycated haemoglobin; HDL-c, high-density lipoprotein cholesterol; LDL-c, low-density lipoprotein cholesterol.

## Data Availability

The datasets used and/or analyzed during the current study are available from the corresponding author on reasonable request.

## Supplementary Materials

The following are available online at https://www.mdpi.com/2072-6643/13/2/326/s1, Table S1: Genotye and allele frequencies of the single nucleotide polymorphisms. Table S2: Association of vitamin D-GRS with 25-hydroxyvitamin D concentrations and clinical and biochemical measurements. Table S3: Interactions of metabolic-GRS with dietary factors on clinical and biochemical measurements.

## Abbreviations

T2D	type 2 diabetes
WC	waist circumference
BMI	body mass index
BFP	body fat percentage
HbA1c	glycated haemoglobin
LDL-c	low density lipoprotein cholesterol
HDL-c	high density lipoprotein cholesterol
*DHCR7*	7-dehydrocholesterol reductase
*CYP2R1*	25-hydroxylase
*CYP24A1*	24-hydroxylase
*DBP*	vitamin D binding protein
*GC*	group-specific component
*CASR*	calcium sensing receptor
*FTO*	fat mass and obesity-associated gene
*TCF7L2*	transcription factor 7-like 2 gene
*MC4R*	melanocortin 4 receptor gene
*KCNQ1*	potassium voltage-gated channel subfamily Q member 1
*CDKN2A*/*B*	cyclin dependent kinase inhibitor 2A/B
GRS	genetic risk score
SNP	single nucleotide polymorphism
MR	Mendelian Randomization
GWAS	genome wide association studies
MINANG	Minangkabau Indonesia study on nutrition and genetics
WHO	World Health Organization
FFQ	food frequency questionnaire
HWE	Hardy Weinberg equilibrium
SD	standard deviation
ELISA	enzyme-linked immunosorbent assay
SQ-FFQ	semi-quantitative food frequency questionnaire

## References

[1] Amrein K., Scherkl M., Hoffmann M., Neuwersch-Sommeregger S., Köstenberger M., Tmava Berisha A., Martucci G., Pilz S., Malle O. (2020). Vitamin D deficiency 2.0: An update on the current status worldwide. Eur. J. Clin. Nutr..

[2] Alathari B.E., Sabta A.A., Kalpana C.A., Vimaleswaran K.S. (2020). Vitamin D pathway-related gene polymorphisms and their association with metabolic diseases: A literature review. J. Diabetes Metab. Disord..

[3] Jiang X., Kiel D.P., Kraft P. (2019). The genetics of vitamin D. Bone.

[4] Been L.F., Ralhan S., Wander G.S., Mehra N.K., Singh J., Mulvihill J.J., Aston C.E., Sanghera D.K. (2011). Variants in KCNQ1 increase type II diabetes susceptibility in South Asians: A study of 3310 subjects from India and the US. BMC Med. Genet..

[5] Bego T., Čaušević A., Dujić T., Malenica M., Velija-Asimi Z., Prnjavorac B., Marc J., Nekvindová J., Palička V., Semiz S. (2019). Association of FTO Gene Variant (rs8050136) with Type 2 Diabetes and Markers of Obesity, Glycaemic Control and Inflammation. J. Med. Biochem..

[6] Chidambaram M., Liju S., Saboo B., Sathyavani K., Viswanathan V., Pankratz N., Gross M., Mohan V., Radha V. (2016). Replication of genome-wide association signals in Asian Indians with early-onset type 2 diabetes. Acta Diabetol..

[7] Chris A., Narila N., Flori S., Risfy P., Intan A., Zeti H., Witri A., Hari H., Nouval S., Endah W. (2019). Preliminary study: Identification of dna variation with snp numbers rs1137101 and rs8050136 in patient’s type 2 diabetes mellitus at salsabila clinic bogor—Indonesia. Asian J. Microbiol. Biotechnol. Environ. Sci..

[8] Gupta V., Khadgawat R., Ng H.K.T., Walia G.K., Kalla L., Rao V.R., Sachdeva M.P. (2012). Association of TCF7L2 and ADIPOQ with body mass index, waist-hip ratio, and systolic blood pressure in an endogamous ethnic group of India. Genet. Test. Mol. Biomark..

[9] Prakash J., Srivastava N., Awasthi S., Agarwal C., Natu S., Rajpal N., Mittal B. (2012). Association of PPAR-γ gene polymorphisms with obesity and obesity-associated phenotypes in north indian population. Am. J. Hum. Biol..

[10] Srivastava A., Mittal B., Prakash J., Narain V.S., Natu S.M., Srivastava N. (2014). Evaluation of MC4R [rs17782313, rs17700633], AGRP [rs3412352] and POMC [rs1042571] Polymorphisms with Obesity in Northern India. Oman Med. J..

[11] Chauhan G., Spurgeon C.J., Tabassum R., Bhaskar S., Kulkarni S.R., Mahajan A., Chavali S., Kumar M.V.K., Prakash S., Dwivedi O.P. (2010). Impact of common variants of PPARG, KCNJ11, TCF7L2, SLC30A8, HHEX, CDKN2A, IGF2BP2, and CDKAL1 on the risk of type 2 diabetes in 5,164 Indians. Diabetes.

[12] Vimaleswaran K.S., Loos R.J. (2010). Progress in the genetics of common obesity and type 2 diabetes. Expert Rev. Mol. Med..

[13] Parrillo L., Spinelli R., Nicolò A., Longo M., Mirra P., Raciti G.A., Miele C., Beguinot F. (2019). Nutritional Factors, DNA Methylation, and Risk of Type 2 Diabetes and Obesity: Perspectives and Challenges. Int. J. Mol. Sci..

[14] Angkurawaranon C., Jiraporncharoen W., Chenthanakij B., Doyle P., Nitsch D. (2014). Urban environments and obesity in southeast Asia: A systematic review, meta-analysis and meta-regression. PLoS ONE.

[15] Aji A.S., Erwinda E., Rasyid R., Yusrawati Y., Malik S.G., Alathari B., Lovegrove J.A., Lipoeto N.I., Vimaleswaran K.S. (2020). A genetic approach to study the relationship between maternal Vitamin D status and newborn anthropometry measurements: The Vitamin D pregnant mother (VDPM) cohort study. J. Diabetes Metab. Disord..

[16] Fajarini I.A., Sartika R.A.D. (2019). Obesity as Type 2 Diabetes Common Comorbidity: Study of Type 2 Diabetes Patients’ Eating Behaviour and Other Determinants in Jakarta, Indonesia. Kesmas Natl. Public Health J..

[17] Harbuwono D.S., Pramono L.A., Yunir E., Subekti I. (2018). Obesity and central obesity in Indonesia: Evidence from a national health survey. Med. J. Indones.

[18] Saeedi P., Petersohn I., Salpea P., Malanda B., Karuranga S., Unwin N., Colagiuri S., Guariguata L., Motala A.A., Ogurtsova K. (2019). Global and regional diabetes prevalence estimates for 2019 and projections for 2030 and 2045: Results from the International Diabetes Federation Diabetes Atlas, 9(th) edition. Diabetes Res. Clin. Pract..

[19] Arifin B., van Asselt A.D.I., Setiawan D., Atthobari J., Postma M.J., Cao Q. (2019). Diabetes distress in Indonesian patients with type 2 diabetes: A comparison between primary and tertiary care. BMC Health Serv. Res..

[20] Ligita T., Wicking K., Francis K., Harvey N., Nurjannah I. (2019). How people living with diabetes in Indonesia learn about their disease: A grounded theory study. PLoS ONE.

[21] Surendran S., Aji A.S., Ariyasra U., Sari S.R., Malik S.G., Tasrif N., Yani F.F., Lovegrove J.A., Sudji I.R., Lipoeto N.I. (2019). A nutrigenetic approach for investigating the relationship between vitamin B12 status and metabolic traits in Indonesian women. J. Diabetes Metab. Disord..

[22] Stark A. (2013). The Matrilineal System of the Minangkabau and its Persistence Throughout History: A Structural Perspective. Southeast Asia Multidiscip. J..

[23] Dudbridge F. (2016). Polygenic Epidemiology. Genet. Epidemiol..

[24] Hüls A., Krämer U., Carlsten C., Schikowski T., Ickstadt K., Schwender H. (2017). Comparison of weighting approaches for genetic risk scores in gene-environment interaction studies. BMC Genet..

[25] Vimaleswaran K.S. (2020). A nutrigenetics approach to study the impact of genetic and lifestyle factors on cardiometabolic traits in various ethnic groups: Findings from the GeNuIne Collaboration. Proc. Nutr. Soc..

[26] Vimaleswaran K.S. (2017). Gene–nutrient interactions on metabolic diseases: Findings from the GeNuIne Collaboration. Nutr. Bull..

[27] Pan W.H., Yeh W.T. (2008). How to define obesity? Evidence-based multiple action points for public awareness, screening, and treatment: An extension of Asian-Pacific recommendations. Asia Pac. J. Clin. Nutr..

[28] Lipoeto N.I., Agus Z., Oenzil F., Wahlqvist M., Wattanapenpaiboon N. (2004). Dietary intake and the risk of coronary heart disease among the coconut-consuming Minangkabau in West Sumatra, Indonesia. Asia Pac. J. Clin. Nutr..

[29] Elkum N., Alkayal F., Noronha F., Ali M.M., Melhem M., Al-Arouj M., Bennakhi A., Behbehani K., Alsmadi O., Abubaker J. (2014). Vitamin D insufficiency in Arabs and South Asians positively associates with polymorphisms in GC and CYP2R1 genes. PLoS ONE.

[30] Foucan L., Vélayoudom-Céphise F.-L., Larifla L., Armand C., Deloumeaux J., Fagour C., Plumasseau J., Portlis M.-L., Liu L., Bonnet F. (2013). Polymorphisms in GC and NADSYN1 Genes are associated with vitamin D status and metabolic profile in Non-diabetic adults. BMC Endocr. Disord..

[31] Grzegorzewska A.E., Bednarski D., Świderska M., Mostowska A., Jagodziński P.P. (2018). The Calcium-Sensing Receptor Gene Polymorphism rs1801725 and Calcium-Related Phenotypes in Hemodialysis Patients. Kidney Blood Press. Res..

[32] Grzegorzewska A.E., Frycz B.A., Świderska M., Niepolski L., Mostowska A., Jagodziński P.P. (2019). Calcium-sensing receptor gene (CASR) polymorphisms and CASR transcript level concerning dyslipidemia in hemodialysis patients: A cross-sectional study. BMC Nephrol..

[33] Kwak S.Y., Yongjoo Park C., Jo G., Yoen Kim O., Shin M.J. (2018). Association among genetic variants in the vitamin D pathway and circulating 25-hydroxyvitamin D levels in Korean adults: Results from the Korea National Health and Nutrition Examination Survey 2011–2012. Endocr. J..

[34] Rooney M.R., Pankow J.S., Sibley S.D., Selvin E., Reis J.P., Michos E.D., Lutsey P.L. (2016). Serum calcium and incident type 2 diabetes: The Atherosclerosis Risk in Communities (ARIC) study. Am. J. Clin. Nutr..

[35] Wang T.J., Zhang F., Richards J.B., Kestenbaum B., van Meurs J.B., Berry D., Kiel D.P., Streeten E.A., Ohlsson C., Koller D.L. (2010). Common genetic determinants of vitamin D insufficiency: A genome-wide association study. Lancet.

[36] Xu X., Mao J., Zhang M., Liu H., Li H., Lei H., Han L., Gao M. (2015). Vitamin D Deficiency in Uygurs and Kazaks Is Associated with Polymorphisms in CYP2R1 and DHCR7/NADSYN1 Genes. Med. Sci. Monit. Int. Med. J. Exp. Clin. Res..

[37] Zhang Y., Wang X., Liu Y., Qu H., Qu S., Wang W., Ren L. (2012). The GC, CYP2R1 and DHCR7 genes are associated with vitamin D levels in northeastern Han Chinese children. Swiss Med. Wkly..

[38] Alathari B.E., Bodhini D., Jayashri R., Lakshmipriya N., Shanthi Rani C.S., Sudha V., Lovegrove J.A., Anjana R.M., Mohan V., Radha V. (2020). A Nutrigenetic Approach to Investigate the Relationship between Metabolic Traits and Vitamin D Status in an Asian Indian Population. Nutrients.

[39] Apalasamy Y.D., Ming M.F., Rampal S., Bulgiba A., Mohamed Z. (2013). Association of melanocortin-4 receptor gene polymorphisms with obesity-related parameters in Malaysian Malays. Ann. Hum. Biol..

[40] Chu A.Y., Workalemahu T., Paynter N.P., Rose L.M., Giulianini F., Tanaka T., Ngwa J.S., Qi Q., Curhan G.C., Rimm E.B. (2013). Novel locus including FGF21 is associated with dietary macronutrient intake. Hum. Mol. Genet..

[41] Vasan S.K., Karpe F., Gu H.F., Brismar K., Fall C.H., Ingelsson E., Fall T. (2014). FTO genetic variants and risk of obesity and type 2 diabetes: A meta-analysis of 28,394 Indians. Obesity.

[42] Zhao Q., Xiao J., He J., Zhang X., Hong J., Kong X., Mills K.T., Weng J., Jia W., Yang W. (2014). Cross-Sectional and Longitudinal Replication Analyses of Genome-Wide Association Loci of Type 2 Diabetes in Han Chinese. PLoS ONE.

[43] Ross A.C., Manson J.E., Abrams S.A., Aloia J.F., Brannon P.M., Clinton S.K., Durazo-Arvizu R.A., Gallagher J.C., Gallo R.L., Jones G. (2011). The 2011 Report on Dietary Reference Intakes for Calcium and Vitamin D from the Institute of Medicine: What Clinicians Need to Know. J. Clin. Endocrinol. Metab..

[44] Abbas M.A. (2017). Physiological functions of Vitamin D in adipose tissue. J. Steroid Biochem. Mol. Biol..

[45] Vimaleswaran K.S., Berry D.J., Lu C., Tikkanen E., Pilz S., Hiraki L.T., Cooper J.D., Dastani Z., Li R., Houston D.K. (2013). Causal relationship between obesity and vitamin D status: Bi-directional Mendelian randomization analysis of multiple cohorts. PLoS Med..

[46] Ahn J., Yu K., Stolzenberg-Solomon R., Simon K.C., McCullough M.L., Gallicchio L., Jacobs E.J., Ascherio A., Helzlsouer K., Jacobs K.B. (2010). Genome-wide association study of circulating vitamin D levels. Hum. Mol. Genet..

[47] Hiraki L.T., Major J.M., Chen C., Cornelis M.C., Hunter D.J., Rimm E.B., Simon K.C., Weinstein S.J., Purdue M.P., Yu K. (2013). Exploring the Genetic Architecture of Circulating 25-Hydroxyvitamin, D. Genet. Epidemiol..

[48] Jiang X., O’Reilly P.F., Aschard H., Hsu Y.H., Richards J.B., Dupuis J., Ingelsson E., Karasik D., Pilz S., Berry D. (2018). Genome-wide association study in 79,366 European-ancestry individuals informs the genetic architecture of 25-hydroxyvitamin D levels. Nat. Commun..

[49] Sapkota B.R., Hopkins R., Bjonnes A., Ralhan S., Wander G.S., Mehra N.K., Singh J.R., Blackett P.R., Saxena R., Sanghera D.K. (2016). Genome-wide association study of 25(OH) Vitamin D concentrations in Punjabi Sikhs: Results of the Asian Indian diabetic heart study. J. Steroid Biochem. Mol. Biol..

[50] Vimaleswaran K.S., Cavadino A., Berry D.J., Whittaker J.C., Power C., Jarvelin M.R., Hypponen E. (2013). Genetic association analysis of vitamin D pathway with obesity traits. Int. J. Obes. (Lond.).

[51] Narvaez C.J., Matthews D., Broun E., Chan M., Welsh J. (2009). Lean phenotype and resistance to diet-induced obesity in vitamin D receptor knockout mice correlates with induction of uncoupling protein-1 in white adipose tissue. Endocrinology.

[52] Wong K.E., Kong J., Zhang W., Szeto F.L., Ye H., Deb D.K., Brady M.J., Li Y.C. (2011). Targeted expression of human vitamin D receptor in adipocytes decreases energy expenditure and induces obesity in mice. J. Biol. Chem..

[53] Usfar A.A., Fahmida U. (2011). Do Indonesians follow its Dietary Guidelines?: Evidence related to food consumption, healthy lifestyle, and nutritional status within the period 2000–2010. Asia Pac. J. Clin. Nutr..

[54] Ministry of Health Republic of Indonesia (2019). The Indonesian Dietary Recommendation (AKG—Angka Kecukupan Gizi).

[55] Sartorius K., Sartorius B., Madiba T.E., Stefan C. (2018). Does high-carbohydrate intake lead to increased risk of obesity? A systematic review and meta-analysis. BMJ Open.

[56] Santos F.L., Esteves S.S., da Costa Pereira A., Yancy W.S., Nunes J.P. (2012). Systematic review and meta-analysis of clinical trials of the effects of low carbohydrate diets on cardiovascular risk factors. Obes. Rev..

[57] Unwin D.J., Tobin S.D., Murray S.W., Delon C., Brady A.J. (2019). Substantial and Sustained Improvements in Blood Pressure, Weight and Lipid Profiles from a Carbohydrate Restricted Diet: An Observational Study of Insulin Resistant Patients in Primary Care. Int. J. Environ. Res. Public Health.

[58] Pittas A.G., Lau J., Hu F.B., Dawson-Hughes B. (2007). The role of vitamin D and calcium in type 2 diabetes. A systematic review and meta-analysis. J. Clin. Endocrinol. Metab..

[59] Newton A.L., Hanks L.J., Ashraf A.P., Williams E., Davis M., Casazza K. (2012). Macronutrient intake influences the effect of 25-hydroxy-vitamin d status on metabolic syndrome outcomes in african american girls. Cholesterol.

[60] Linnemann A.K., Baan M., Davis D.B. (2014). Pancreatic β-cell proliferation in obesity. Adv. Nutr..

[61] Lipoeto N.I., Agus Z., Oenzil F., Masrul M., Wattanapenpaiboon N., Wahlqvist M.L. (2001). Contemporary Minangkabau food culture in West Sumatra, Indonesia. Asia Pac. J. Clin. Nutr..

[62] Adab P., Pallan M., Whincup P.H. (2018). Is BMI the best measure of obesity?. BMJ.

[63] Carpenter C.L., Yan E., Chen S., Hong K., Arechiga A., Kim W.S., Deng M., Li Z., Heber D. (2013). Body fat and body-mass index among a multiethnic sample of college-age men and women. J. Obes..

[64] Alsulami S., Aji A.S., Ariyasra U., Sari S.R., Tasrif N., Yani F.F., Lovegrove J.A., Sudji I.R., Lipoeto N.I., Vimaleswaran K.S. (2020). Interaction between the genetic risk score and dietary protein intake on cardiometabolic traits in Southeast Asian. Genes Nutr..

