# Supplementary material for: Interaction between Vitamin D-Related Genetic Risk Score and Carbohydrate Intake on Body Fat Composition: A Study in Southeast Asian Minangkabau Women
Source: Nutrients. 2021 Jan 23;13(2):326. doi: 10.3390/nu13020326 (PMC7911469; doi:10.3390/nu13020326)
Supplement: Supplementary file 1 [file nutrients-13-00326-s001.pdf]

Supplementary Table S1. Genotypic and allelic frequencies of the single nucleotide polymorphisms that were used to create the genetic risk score.

Gene Symbols	Single Nucleotide Polymorphisms	Nucleotide Change	Total	Common Homozygotes	Heterozygotes	Rare Homozygotes	Hardy Weinberg Equilibrium *p* Value	Minor Allele Frequency	Minor Allele and Frequency in East Asians (dbSNP* -EAS)	Minor Allele and Frequency in Europeans (dbSNP* -EUR)	Minor Allele and Frequency in South Asian (dbSNP* -SAS)
FTO	rs8050136	A/C	115	CC: 69	CA: 39	AA: 7	0.64	A= 0.23	A (0.17)	A (0.41)	A (0.29)
	rs9939609	A/T	116	TT: 70	TA: 39	AA: 7	0.62	A= 0.23	A (0.17)	A (0.41)	A (0.29)
	rs10163409	T/A	117	AA: 106	TA: 11	TT: 0	0.59	T= 0.05	T (0.03)	T (0.31)	T (0.20)
TCF7L2	rs12255372	T/G	117	GG: 97	TG: 20	TT: 0	0.31	T= 0.09	T (0.01)	T (0.29)	T (0.22)
	rs7903146	T/C	116	CC: 95	TC: 21	TT: 0	0.28	T= 0.09	T (0.02)	T (0.32)	T (0.30)
MC4R	rs17782313	C/T	117	TT: 89	TC: 26	CC: 2	0.95	C= 0.13	C (0.19)	C (0.24)	C (0.32)
	rs2229616	A/G	117	GG: 116	GA: 1	AA: 0	0.96	A= 0.00	A (0.02)	A (0.01)	A (0.03)
KCNQ1	rs2237895	C/A	115	AA: 58	CA: 46	CC: 11	0.67	C= 0.30	C (0.33)	C (0.42)	C (0.40)
	rs2237892	T/C	117	CC: 44	TC: 53	TT: 20	0.56	T= 0.40	T (0.36)	T (0.06)	T (0.01)
CDKN2A/B	rs10811661	C/T	116	TT: 50	CT: 57	CC: 9	0.19	C= 0.32	C (0.44)	C (0.17)	C (0.13)
DHCR7	rs12785878	T/G	117	GG: 73	TG: 36	TT: 8	0.23	T= 0.22	T (0.38)	G (0.30)	T (0.15)
CYP2R1	rs12794714	A/G	117	GG: 62	GA: 50	AA: 5	0.19	A= 0.26	A (0.37)	A (0.45)	A (0.44)
CYP24A1	rs6013897	A/T	116	TT: 54	TA: 48	AA: 14	0.51	A= 0.33	A (0.15)	A (0.24)	A (0.33)
GC	rs2282679	C/A	115	AA: 77	CA: 33	CC: 5	0.55	C= 0.19	C (0.26)	C (0.25)	C (0.30)
CASR	rs1801725	T/G	117	GG: 96	TG: 19	TT: 2	0.36	T= 0.10	T (0.04)	T (0.15)	T (0.21)

*dbSNP database: https://www.ncbi.nlm.nih.gov/snp/ [1].

Supplementary Table S2. Association of vitamin D-GRS with 25-hydroxyvitamin D concentrations and clinical and biochemical measurements.

Clinical and Biochemical Parameters	GRS ≤ 2		GRS > 2		
	n	Mean±SD	*n*	Mean±SD	*p* Value
25-hydroxyvitamin D (ng/ml)	67	1.24± 0.20	43	1.25± 0.20	0.93
BMI (kg/m^2)	67	25.05± 4.12	43	25.41± 4.47	0.70
WC (cm)	67	1.90± 0.14	43	1.92± 0.07	0.59
BFP (%)	67	1.54±0.09	43	1.56±0.10	0.30
Glucose (mg/dl)	67	1.96± 0.08	43	1.96± 0.06	0.99
HbA1c (ng/ml)	67	2.67± 0.27	43	2.74± 0.32	0.19
Fasting Insulin (nmlU/L)	67	4.39± 0.23	43	4.46± 0.28	0.15
Total Cholesterol (mg/dl)	67	2.32± 0.09	43	2.30± 0.09	0.12
HDL Cholesterol (mg/dl)	67	1.77± 0.08	43	1.77± 0.07	1.00
LDL Cholesterol (mg/dl)	67	2.10± 0.18	43	2.05± 0.17	0.16
Serum Triglycerides (mg/dl)	67	2.00± 0.1.8	43	2.00± 0.1.8	0.79

All variables were log transformed except for BMI values which were in normal distribution. All associations were adjusted for location, age and BMI (except BMI which was not adjusted for when the outcome was BMI). Abbreviations: GRS: genetic risk score, BMI: body mass index, WC: waist circumference, BFP: body fat percentage, HbA1c: glycated haemoglobin, HDL: high density lipoprotein, LDL: low density lipoprotein.

Supplementary Table S3. Interactions between metabolic-GRS and dietary factors on clinical and biochemical measurements.

Clinical and Biochemical Parameters	Carbohydrate (g)	Protein (g)	Fat (g)	Fibre (g)
	$p_{interaction}$	$p_{interaction}$	$p_{interaction}$	$p_{interaction}$
25-hydroxyvitamin D (ng/ml)	0.83	0.93	1.00	0.68
BMI (kg/m^2)	0.34	0.91	0.34	0.56
WC (cm)	0.58	0.10	0.15	0.47
BFP (%)	0.32	0.70	0.96	0.84
Glucose (mg/dl)	0.36	0.56	0.70	0.83
HbA1c (ng/ml)	0.78	0.68	0.78	0.53
Fasting Insulin (nmlU/L)	0.63	0.22	0.44	0.44
Total Cholesterol (mg/dl)	0.17	0.52	0.32	0.21
HDL Cholesterol (mg/dl)	0.18	0.52	0.70	0.65
LDL Cholesterol (mg/dl)	0.34	0.95	0.85	0.55
Serum Triglycerides (mg/dl)	0.95	0.54	0.77	0.38

All variables were log transformed except for BMI values which had a normal distribution. All interactions were adjusted for age, location, total energy intake, and BMI (except BMI which was not adjusted for when the outcome was BMI). Abbreviations: BMI: body mass index, WC: waist circumference, BFP: body fat percentage, HbA1c: glycated haemoglobin, HDL: high density lipoprotein, LDL: low density lipoprotein.

Refrences

1. National Center for Biotechnology Information. https://www.ncbi.nlm.nih.gov/snp/.
